# CXXC4 activates apoptosis through up-regulating GDF15 in gastric cancer

**DOI:** 10.18632/oncotarget.21581

**Published:** 2017-10-06

**Authors:** Mengjiao Han, Dongjun Dai, Neelum Aziz Yousafzai, Faliang Wang, Hanying Wang, Qiying Zhou, Haiqi Lu, Wenxia Xu, Lifeng Feng, Hongchuan Jin, Xian Wang

**Affiliations:** ^1^ Department of Medical Oncology, Sir Run Run Shaw Hospital, Medical School of Zhejiang University, Hangzhou, China; ^2^ Labortaory of Cancer Biology, Key Laboratory of Biotherapy in Zhejiang, Sir Run Run Shaw Hospital, Medical School of Zhejiang University, Hangzhou, China

**Keywords:** CXXC4, GDF15, apoptosis, gastric cancer

## Abstract

Worldwide, gastric cancer is one of the most fatal cancers. Epigenetic alterations in gastric cancer play important roles in silencing of tumor suppressor genes. We previously found that CXXC finger protein 4 (CXXC4) was a novel tumor suppressor in gastric cancer. In this report, we demonstrated that CXXC4 inhibited growth of gastric cancer cells as a pro-apoptotic factor. This inhibition could be reversed by the pan-caspase inhibitor called Z-VAD-FMK. However, CXXC4 with mutations in its DNA binding domain failed to induce apoptosis. Growth differentiation factor 15 (GDF15) was identified as one of potential targets responsible for CXXC4-induced apoptosis. CXXC4 activated GDF15 transcription through enhancing the interaction of transcription factor Sp1 with GDF15 promoter. In summary, the nuclear protein CXXC4 activated apoptosis in gastric cancer through up-regulating its novel potential downstream target GDF15. GDF15 might be a promising target for clinical treatment of gastric cancer with CXXC4 deficiency.

## INTRODUCTION

Despite recent advances in diagnosis and treatment, gastric cancer remains the fourth most common cancer worldwide [1] and the second leading cause of cancer deaths [2–4]. Genetic and epigenetic alterations play significant roles in gastric carcinogenesis [5]. As a result, aberrant activation of oncogenes and inactivation of tumor suppressor genes eventually altered signaling pathways critical for cell proliferation, differentiation, cell cycle and cell fate decision in gastric cancer [5–8].

We recently found that tumor suppressor CXXC finger protein 4 (CXXC4) was downregulated in gastric cancer and its downregulation was associated with a poor prognosis in gastric cancer patients [9]. It was directly regulated by EZH2 and functioned to negatively regulate Wnt/β-catenin and Ras/MAPK signaling [9–12]. However, CXXC4 was named after its CXXC domain which was well-known for the DNA-binding capability, indicating that CXXC4 might play a role in the regulation of genome integrity or function [13–15]. Indeed, we found in this report that CXXC4 located in the nucleus and was able to stimulate the transcription of GDF15 (growth differentiation factor 15) to activate apoptosis.

GDF15 is a divergent member of the transforming growth factor-beta (TGF-β) superfamily that exerts complex effects on several cellular pathophysiology conditions including stress responses, bone formation, ischemia, and cancers [16, 17]. Previous evidence demonstrated that GDF15 had both anti-tumorigenic and pro-tumorigenic activities. For example, GDF15 expression could be activated by tumor suppressor genes p53, EGR-1, and GSK-3β to exhibit pro-apoptotic activities in colorectal, lung and other cancers [18–21]. Moreover, GDF15 could induce apoptosis by upregulating death receptor-4 (DR4) and DR-5 expression in gastric cancer cells [22]. In contrast, the aberrant increase of secreted GDF15 in serum correlated with poor prognosis in several cancers such as prostate cancer, breast cancer and gastric cancer [23]. Therefore, the role of GDF15 may vary from different cancer types [16]. However, the exact mechanism of GDF15 regulation in gastric carcinogenesis remains rarely understood.

## RESULTS

### CXXC4 inhibited cell growth by activating apoptosis

**Figure 1:**
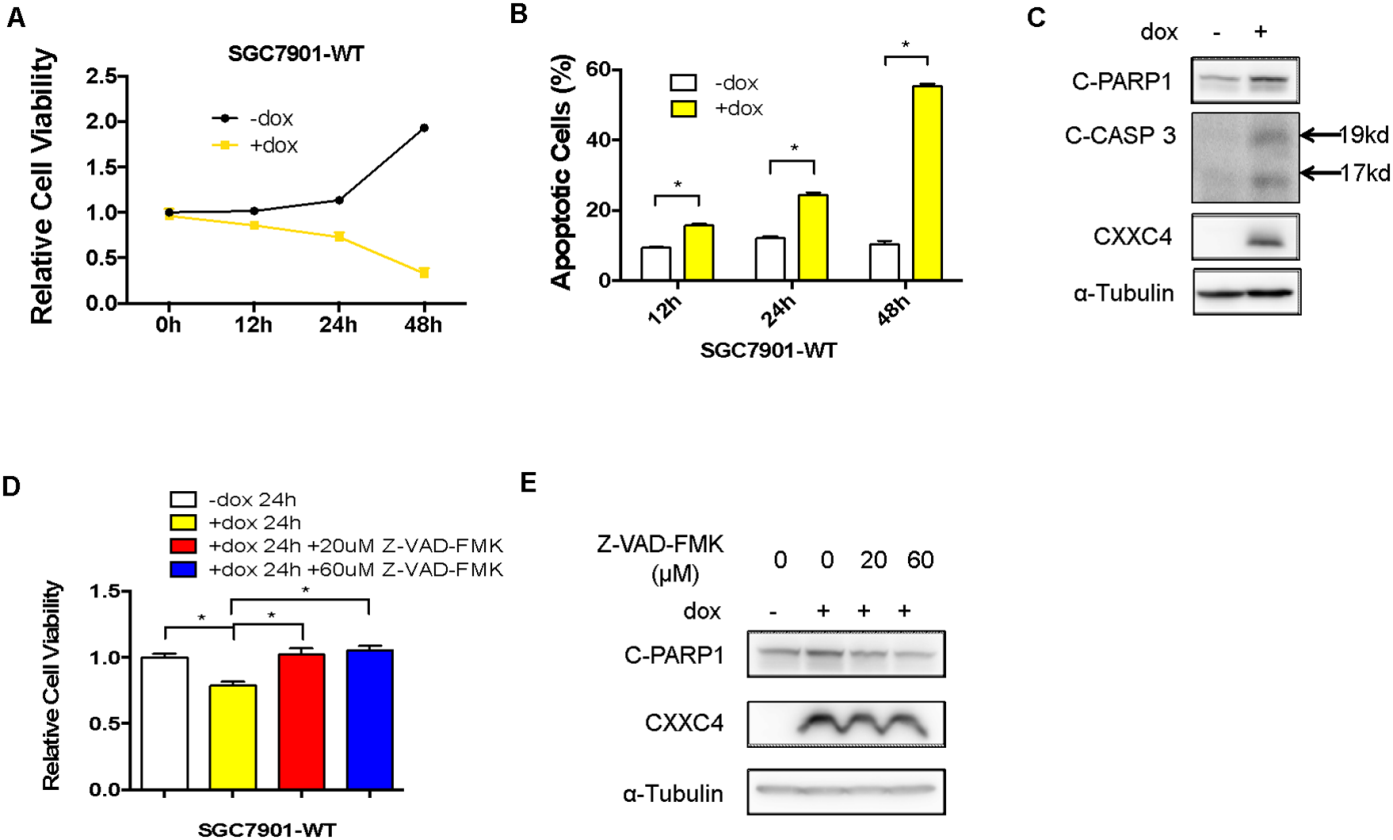
CXXC4 inhibited cell growth by activating apoptosis **(A)** The viability of SGC7901-WT cells treated with Dox on the concentration of 0.5μg/ml after different time was analyzed by MTS assay. **(B)** The apoptosis of SGC7901-WT cells treated with Dox on the concentration of 0.5 μg/ml after different time was determined by flow cytometry. **(C)** SGC7901-WT cells were treated with Dox (0.5μg/ml) for 24 hours and collected to determine the protein level of cleaved PARP1, cleaved Caspase 3 and CXXC4 by Western blotting. **(D)** The viability of SGC7901-WT cells treated with Dox and the pan-caspase inhibitor Z-VAD-FMK was analyzed by MTS assay. **(E)** The effect of the pan-caspase inhibitor Z-VAD-FMK on SGC7901-WT cells pre-treated with Dox was determined by Western blotting. (*P<0.05).

We have found previously that CXXC4 served as a tumor suppressor gene in human gastric cancer [24]. To further investigate the functions of CXXC4 in gastric cancer, a stable doxycycline (Dox)-dependent CXXC4 expressing SGC7901 (SGC7901-WT) cell line was established. As expected, the growth of SGC7901-WT cells was remarkably inhibited upon Dox-induced expression of CXXC4 (Figure 1A). Meanwhile, cell apoptosis was significantly increased (Figure 1B and 1C). Z-VAD-FMK, a pan-caspase inhibitor, potently reversed CXXC4-promoted cell apoptosis and growth inhibition (Figure 1D and 1E). Taken together, these results indicated that CXXC4 suppressed cell growth through activating apoptosis.

### CXXC4 localized in the nucleus and activated apoptosis through its DNA binding domain

**Figure 2:**
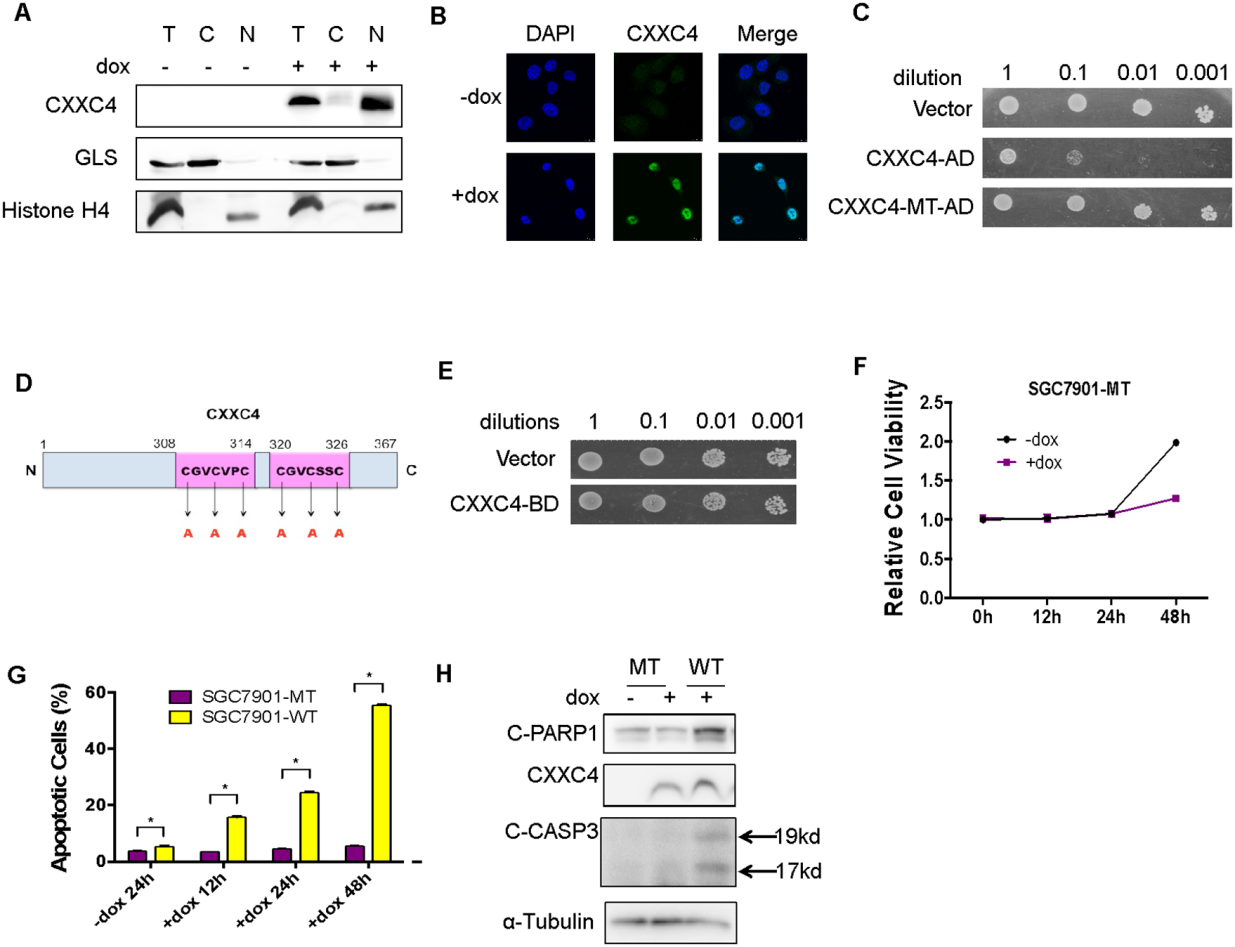
CXXC4 localized in the nucleus and activated apoptosis through its DNA binding domain **(A)** Cytoplasm and nuclear fractions of the SGC7901-WT cells were isolated to detect the location of CXXC4 by Western blotting. **(B)** SGC7901-WT cells treated with or without Dox (0.5μg/ml) for 24h were analyzed by immunofluorescence. DAPI was used to stain the nuclei. **(C)** The proliferation of yeast cells transfected with CXXC4-AD or CXXC4-MT-AD were analyzed compared to the proliferation of yeast cells transfected with empty vector. **(D)** Schematic representation of mutant CXXC4 sites. **(E)** The proliferation of yeast cells transfected with CXXC4-BD was analyzed compared to the proliferation of yeast cells transfected with empty vector. **(F)** The viability of SGC7901-MT cells treated with Dox on the concentration of 0.5μg/ml after different time was analyzed by MTS assay. **(G)** The apoptosis of SGC7901-MT cells treated with Dox on the concentration of 0.5 μg/ml after different time was determined by flow cytometry. **(H)** SGC7901-MT cells were treated with Dox (0.5μg/ml) for 24 hours and collected to determine the protein level of cleaved PARP 1, cleaved Caspase 3 and CXXC4 by Western blotting. (*P <0.05).

CXXC domain has been demonstrated in several proteins characteristic of specific DNA binding activity, indicating that CXXC4 might exert its functions in the nucleus [12]. We therefore investigated the cellular location of CXXC4 and whether it could bind to DNA like other CXXC-containing proteins. Firstly, nuclear/cytoplasmic fractionation of SGC7901-WT cells demonstrated that CXXC4 was mainly located in the nucleus (Figure 2A). Moreover, an immunofluorescence assay was also conducted to further validate the nuclear location of CXXC4 (Figure 2B). Subsequently, a Yeast Two Hybrid system was introduced to evaluate the DNA binding activity of CXXC4. We constructed expression vectors expressing CXXC4-AD (GAL4 activation domain) and CXXC4-BD (GAL4 binding domain) fusion proteins and transformed them into yeast cells, respectively. Interestingly, we found that CXXC4-AD fusion protein could inhibit yeast cell proliferation (CXXC4-WT-AD in Figure 2C). However, it lost its growth inhibitory ability after the disruption of potential DNA-binding sites by mutating all six Cysteines to Alanines in CXXC domain (Figure 2C and 2D). In contrast, CXXC4-BD fusion protein failed to constrain yeast cell proliferation (Figure 2E). To validate this finding in mammalian cells, we expressed CXXC4 mutant with defective CXXC domain in human gastric cancer cells as described in Figure 1 (SGC7901-MT in Figure 2F–2H). In contrast to wild type CXXC4, the CXXC4 mutant was unable to induce growth inhibition (Figure 2F) and apoptosis activation (Figure 2G and 2H). Collectively, these results suggested that CXXC4 had a DNA binding ability to potentially affect the transcription of gene related to apoptosis.

### Identification of GDF15 as a potential target of CXXC4

**Figure 3:**
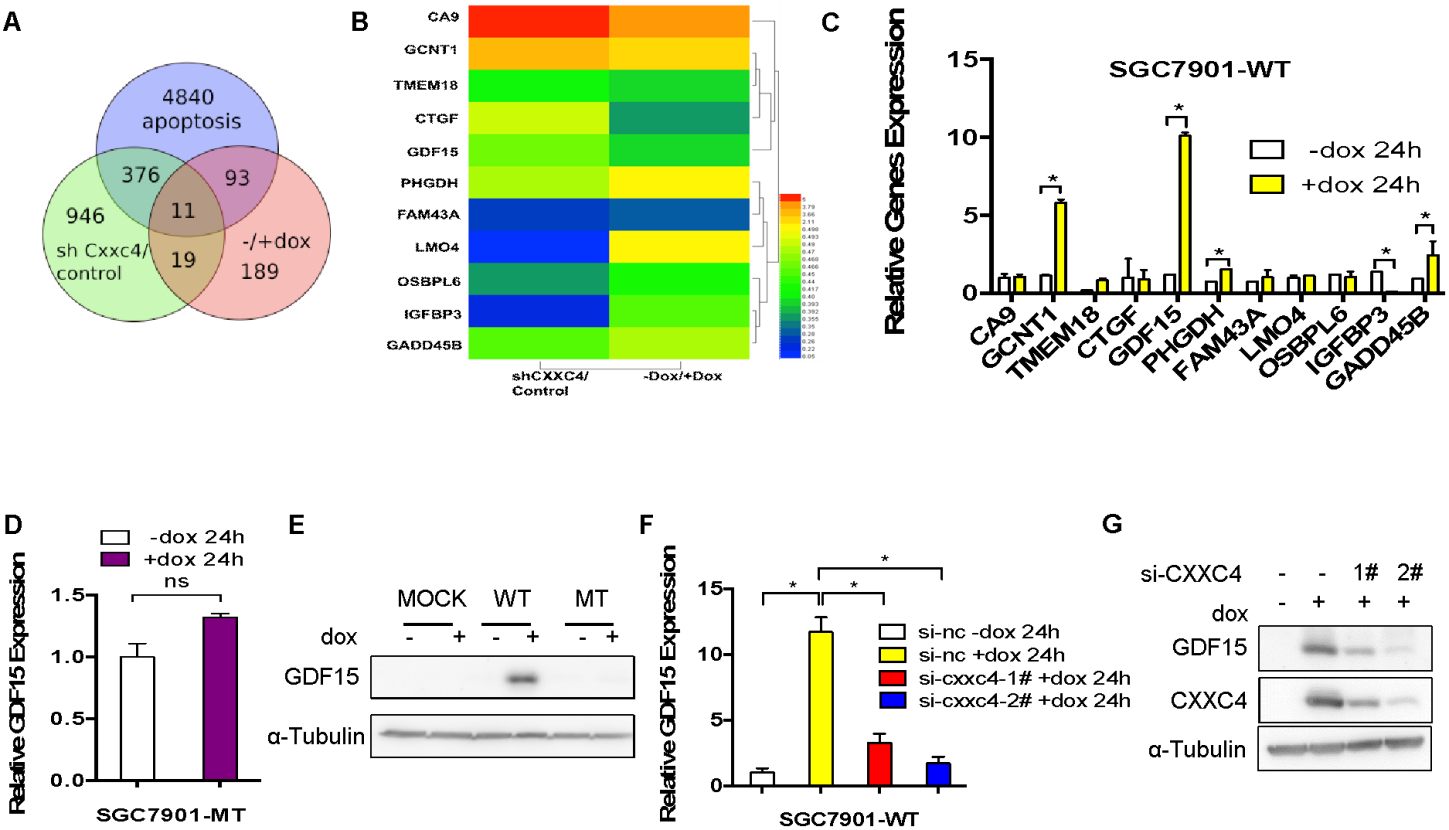
Identification of GDF15 as a potential target of CXXC4 **(A)** Deregulated genes in cells with depletion of CXXC4 or overexpression of CXXC4 and apoptotic relative genes in SGC7901-WT cells were overlapped. **(B)** Deregulation of potential CXXC4 target genes was summarized by heat map. **(C)** The expressions of 11 genes before and after CXXC4 expression were determined by quantitative RT-PCR. **(D)** GDF15 expression before and after CXXC4 expression in SGC7901-MT cells was analyzed by qRT-PCR. **(E)** GDF15 expression in SGC7901-MOCK, WT, MT cells before and after Dox treatment were analyzed by western blotting. **(F)** GDF15 expression before and after CXXC4 depletion in SGC7901-WT cells that were pre-treated with Dox for 24h was analyzed by RT-qPCR. **(G)** The amount of GDF15 in SGC7901-WT cells before and after CXXC4 depletion was determined by western blotting. (*P <0.05).

To further uncover how CXXC4 affects the apoptosis, we analyzed gene expression profiles before and after CXXC4 depletion or overexpression in human gastric cancer cells. Among the 30 genes potentially regulated by CXXC4 (Figure 3A), 11 genes were relative to apoptosis (Figure 3B). We chose GDF15 for further analysis since it was significantly upregulated after the expression of wild CXXC4 rather than mutated CXXC4 (Figure 3C and 3D). Wild type also increased the protein level of GDF15 (Figure 3E). In addition, knocking-down of CXXC4 expression reversed CXXC4-stimulated GDF15 expression at both mRNA and protein levels (Figure 3F and 3G), further supporting the specific regulation of GDF15 expression by CXXC4. Therefore, GDF15 was a novel target directly regulated by CXXC4.

### CXXC4 activated apoptosis through GDF15

**Figure 4:**
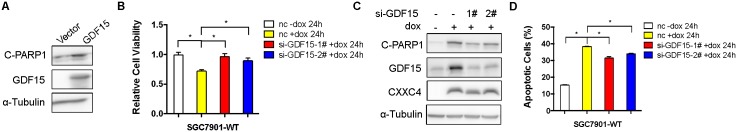
CXXC4 activated apoptosis through GDF15 **(A)** The effect of ectopic GDF15 expression on SGC7901-WT cells was analyzed by western blotting. **(B)** The viability of SGC7901-WT cells that were pre-treated with Dox for 24h after GDF15 depletion was analyzed by MTS assay. **(C)** The levels of cleaved PARP1 and CXXC4 before and after GDF15 depletion were determined by western blotting. **(D)** The apoptosis of SGC7901-WT cells that were pre-treated with Dox for 24h after GDF15 depletion was determined by flow cytometry. (*P <0.05).

As CXXC4 functioned to be a tumor suppressor and GDF15 was a novel CXXC4 downstream target, we further explored the potential tumor suppressing function of GDF15 in gastric cancer. Indeed, overexpression of GDF15 effectively induced the apoptosis in SGC7901 cells (Figure 4A). Furthermore, the growth inhibitory effect of CXXC4 was greatly impaired by the depletion of GDF15 (Figure 4B). Consistently, cell apoptosis was less activated by CXXC4 when GDF15 expression was knocked down (Figure 4C and 4D). In summary, CXXC4 activates apoptosis through upregulating GDF15 expression.

### CXXC4 activated GDF15 transcription through enhancing the interaction of Sp1 with GDF15 promoter

**Figure 5:**
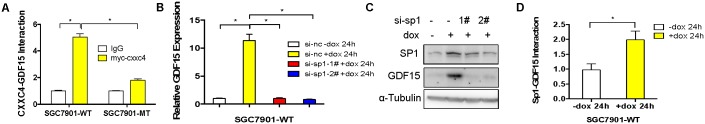
CXXC4 activated GDF15 transcription through enhancing the interaction of Sp1 with GDF15 **(A)** The interaction of CXXC4 with GDF15 promoter in SGC7901-WT cells was analyzed by ChIP qPCR. **(B)** GDF15 expression before and after Sp1 depletion in SGC7901-WT cells that were pre-treated with Dox for 24h was analyzed by RT-qPCR. **(C)** The amount of GDF15 in SGC7901-WT cells before and after Sp1 depletion was determined by western blotting. **(D)** The interaction of Sp1 to GDF15 promoter region in SGC7901-WT cells was analyzed by ChIP followed by qPCR. (*P <0.05).

Next, we further investigated the mechanism how CXXC4 activated GDF15 transcription. Chromatin immunoprecipitation (ChIP) assay revealed that wild type CXXC4 enriched more GDF15 promoter DNA than mutated CXXC4 (Figure 5A), indicating a physical interaction of CXXC domain in CXXC4 with GDF15 promoter. Interestingly, we have found the binding consensus sequence of the transcriptional factor called Specificity protein 1(Sp1) was presented in the GC box located between -133 bp and -41bp of the GDF15 promoter [20, 25–30]. Therefore, we hypothesized that Sp1 could be involved in the transcriptional regulation of GDF15. Indeed, knockdown of Sp1 led to the decreased expression of GDF15 both in mRNA and protein levels (Figure 5B and 5C). ChIP experiment also confirmed the interaction of Sp1 with GDF15 promoter (Figure 5D). Importantly, the binding ability of Sp1 to GDF15 promoter was further enhanced in the presence of CXXC4 (Figure 5D). Therefore, CXXC4 activated GDF15 transcription probably through enhancing the interaction of Sp1 with GDF15.

## DISCUSSION

**Figure 6:**
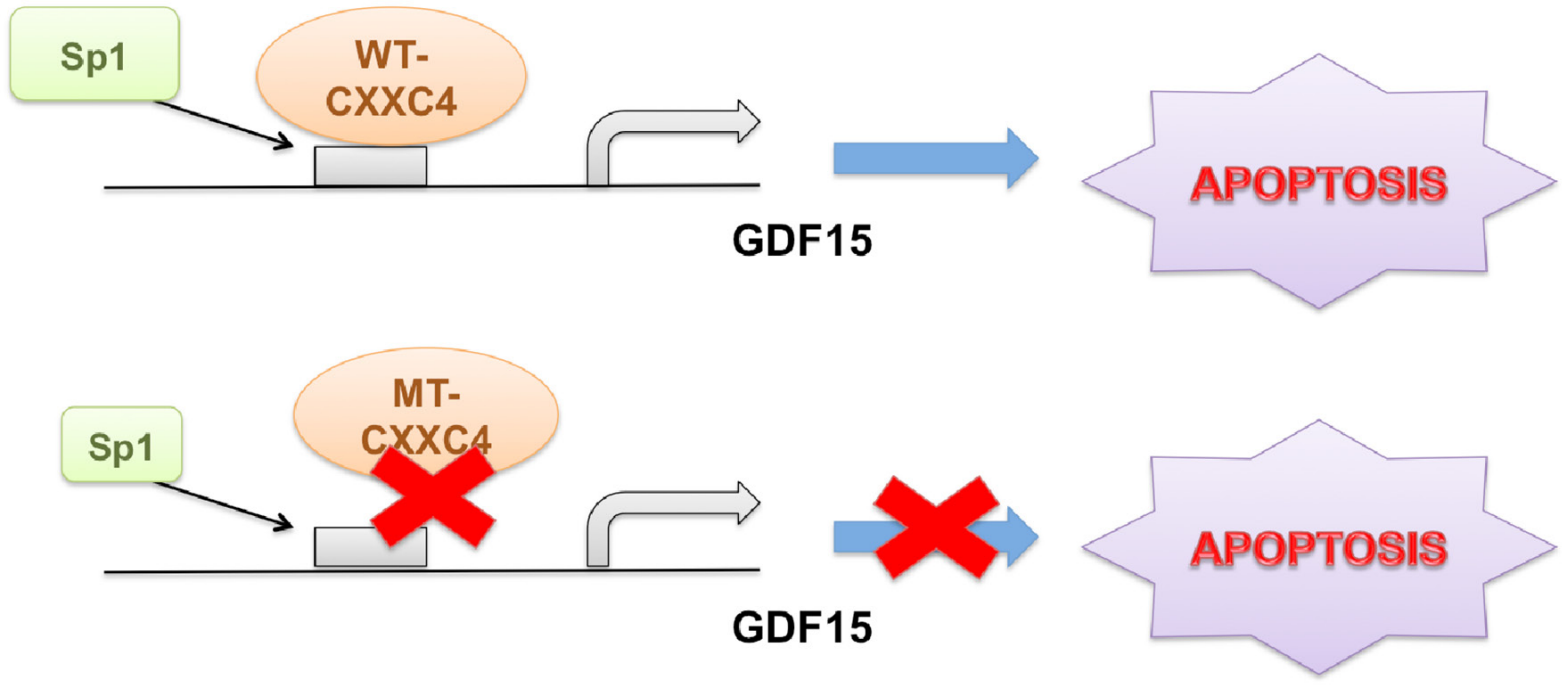
Schematic diagram of CXXC4 activated apoptosis CXXC4 activated GDF15 transcription through enhancing the interaction of Sp1 with GDF15 promoter to induce cell apoptosis in human gastric cancer. While CXXC4 with mutations in its DNA binding domain failed to induce apoptosis through the pathway mentioned above.

Previously, CXXC4 was identified as a tumor suppressor regulated by EZH2 [24, 31]. It functioned to inhibit both Wnt/β-catenin signaling pathway by interacting with Disheveled and MAPK/ERK signaling by binding to MEK1 [9, 11, 24]. By doing so, CXXC4 down-regulation was associated with poor outcome in gastric cancer patients [9, 11, 12, 24, 31–34]. However, given the presence of CXXC domain, CXXC4 might have other functions related with its potential DNA binding capability. For example, CXXC4 was found to downregulate TET2/3 (Ten-Eleven-Translocation 2/3) protein expression and caspase activation in a manner depending on the interaction of its CXXC domain with DNA [12, 15, 33, 35]. In the present study, we found CXXC4 activated the transcription of GDF15 to induce apoptosis in gastric cancer (Figure 6).

CXXC-type zinc finger domain has been shown to be related to DNA and histone modification [13–15]. CXXC4 gene seems to be evolved from chromosomal gene inversion of the ancestral TET2 (tet methylcytosine dioxygenase 2) gene, thus separating the CXXC domain from the catalytic domain. CXXC4 could bind DNA sequences containing unmethylated CpG dinucleotides [12]. We illustrated that CXXC4 was located in the nucleus and activated apoptosis through its CXXC DNA binding domain. We provided the preliminary evidence that GDF15 served as a potential target of CXXC4. After the mutation of all six amino acids (Cysteine to Alanine) in CXXC domain, both growth inhibition and GDF15 induction were impaired. The relevance of DNA methylation/demethylation to CXXC4-activated GDF15 transcription needs to be further clarified.

GDF15 could function both positively and negatively on tumorigenesis and the nature of its effects depends on the cellular context and cell type [16]. Given the results of our present study, it is likely that CXXC4 was a positive regulator of GDF15 and GDF15 also functioned as a suppressor of tumor progression by inducing cell apoptosis in human gastric cancer. These findings are consistent with some reports that showed GDF15 was responsible for Nonsteroidal Anti-inflammatory Drugs (NSAID)-induced apoptosis in gastric cancer cells [22, 36–38] and the expression of GDF15 in cancer tissues was lower than that of normal gastric tissues [39, 40]. However, some studies found that GDF15 expression was up-regulated in gastric cancer patients and its expression was strongly associated with cancer metastasis and invasiveness [23, 41–43]. Nonetheless, our studies clarified a new signaling pathway of CXXC4/GDF15-induced apoptosis in gastric cancer.

Recent studies have shed light on the possible transcription factors important to the regulation of GDF15 transcription, such as p53, EGR-1 and Sp1 [16, 25, 28, 29, 44]. Interestingly, the interaction of Sp1 with GDF15 promoter was enhanced in the presence of CXXC4. However, its remains unknown whether CXXC4 recruits Sp1 to GDF15 promoter or CXXC4 remodels GDF15 promoter from optimal Sp1 interaction. More studies are needed to figure out the underlying mechanisms and validate such a regulation in other cancers.

In summary, CXXC4 stimulated GDF15 transcription via enhancing Sp1 binding to activate apoptosis in human gastric cancer. These findings have potential implications for CXXC4 with respect to its suppressive role on the pathogenesis of gastric cancer. Therefore, our results opened a new avenue for investigating novel functions of CXXC4 relevant to cancer development and provide important information for the management of human gastric cancer.

## MATERIALS AND METHODS

### Cell lines, antibodies and plasmids

All cell lines were cultured in RPMI 1640 medium supplemented with 10% fetal bovine serum and incubated at 5% CO2, 37°C and 95% humidity, unless specifically indicated. Antibody to CXXC4 was purchased from Thermo Fisher (Rockford, IL, USA). Antibodies to Cleaved-PARP1 and Cleaved-Caspase 3 were purchased from Cell Signaling Technology (Boston, MA, USA). Antibody to α-tubulin was purchased from Sigma-Aldrich (St. Louis, MO, USA). Antibody to GLS was purchased from Abgent (San Diego, CA, USA). Antibody to Histone H4 was purchased from Abcam (Cambridge, MA, USA). Antibody to GDF15 was purchased from Abclonal (Cambridge, MA, USA). Antibody to Sp1 was purchased from Santa Cruz (Dallas, TX, USA). Z-VAD-FMK was purchased from Selleckchem (Houston, TX, USA). The GDF15 plasmid was kindly given by professor Maode Lai (Zhejiang University, Hangzhou, China) [45–47].

### Flow cytometry analysis

4×10^5^ cells were cultured overnight in 6-well plates treated with or without doxycycline (Dox) and harvested after 12, 24 or 48 hours. Cell apoptosis was detected with apoptosis kit (FITC Annexin V Apoptosis Detection Kit I, BD Bioscience, Bedford, MA, USA). Briefly, cells were washed twice with cold phosphate buffer saline (PBS) and incubated in 100 μL binding buffer with 5 μL of FITC Annexin V and 5 μL PI for 15 minutes in the dark followed by flow cytometry analysis.

### Cell viability assay

MTS assay was performed with the CellTiter 96 ^®^ Aqueous No-Radioactive Cell Proliferation Assay Kit (Promega, Madison, WI, USA). The cells were seeded into a 96-well plate and cultured overnight before adding Dox or PBS for 12hr, 24hr, 36hr or 48hr, the cell viability was measured following the manufacturer’s instruction.

### Western blotting analysis

Cells treated with various concentrations were collected and lysed in Cytobuster Protein Extraction Reagent (Novagen, Darmstadt, Germany) and protein concentrations were determined by Bio-Rad protein assay kit II (Bio-Rad Laboratories, Hercules, CA, USA). Equal amounts of cellular protein were resolved by SDS-PAGE and transferred to PVDF membrane. The membrane was probed with the indicated primary antibodies, washed with TBS-T (0.01M TRIS-HCl Buffer, 8.8g/L NaCl, 0.1% Tween-20), then incubated with suitable HRP-conjugated second antibodies (Dako, Copenhagen, DK) and visualized with enhanced chemiluminescence (EMD Millipore, Temecula, CA, USA).

### Lentivirus infection

To generate the Dox-inducible CXXC4 expression vector, Myc flag followed by CXXC4 coding sequence was sub-cloned into CV051 vector (Genechem, Shanghai, China) using EcoRI and MluI restriction sites. SGC7901-WT cells were generated by transfecting with vector. SGC7901-MT cells were generated by transfecting with lentivirus containing the mutant CXXC domain (6C to 6A) vector. SGC7901-MOCK cells were generated by transfecting with lentivirus containing the empty vector. After infection, the cells with plasmid described above were selected for 2 weeks with complete RPMI 1640 medium containing puromycin (1 μg/mL).

### Yeast two-hybrid analysis

Full length CXXC4 (CXXC4-WT) or mutant (CXXC4-MT) were inserted into yeast two-hybrid system plasmids pGBKT7 or pGADT7. CXXC4/pGBKT7 or CXXC4/pGADT7 plasmids were transformed into yeast strain Y2HGold respectively according to the manufacturer’s instruction, and transformants were grown on plates with synthetic complete medium without Trp (for CXXC4/pGBKT7) or Leu (for CXXC4/pGADT7) in a 30°C incubator for 3-5 days. Yeast colonies were re-amplified in synthetic complete medium with shaking at 200rpm under 30°C overnight. Finally, a series of 1/10 dilution of yeast cells from an initiation concentration with 1*10^6^ cells/mL were prepared, and 10 μL of each diluted yeast cells were dotted on the suitable plates with the synthetic complete medium. The growth of yeast cells was checked and photographed after 3-5 days incubation.

### RNA extraction and quantitative real-time RT-qPCR

Total RNA was extracted by a TRIzol reagent (Invitrogen, Carlsbad, CA, USA) according to the manufacturer’s instruction. RNA concentrations were quantified by NanoDropTM 2000 (Thermo Scientific, Waltham, MA, USA). Reverse transcription reaction was performed using 2μg of total RNA with Quantscript RT Kit (Tiangen biotechnology, Beijing, China). The mRNA expression level was determined by RT-qPCR using Bestar SybrGreen qPCR mastermix (DBI, Shanghai, China) and LightCycler 480^®^ II Real-Time PCR System (Roche, Basel, CH). Primers used are listed in Table 1.

**Table 1:** Primers and siRNAs used in the study

Primers	
Name	Sequence
GAPDH	F: GGAGTCAACGGATTTGGT
	R: GTGATGGGATTTCCATTGAT
CA9	F: TGACTTCAGCCGCTACTTCC
	R: GTTCAGCTGTAGCCGAGAGT
GCNT1	F: AAGACTCGTGCAGCACATCA
	R: TTATCAGAGCTGCAACGGCA
TMEM18	F: AGAGAGGATTTGGCGCCCT
	R: GTCCAGTCCGTCTGCGTGAG
CTGF	F: TTAGCGTGCTCACTGACCTG
	R: GCCACAAGCTGTCCAGTCTA
GDF15	F: GCAAGAACTCAGGACGGTGA
	R: CGAGGTCGGTGTTCGAATCT
PHGDH	F: GAGCGGGAGCTGGAGAATAC
	R: GGCTGTCACTGATGAGCACT
FAM43A	F: CCGTCAGGGCACCAAGATG
	R: CGGTACACCCAGGCGAAGA
LMO4	F: ATCCACATGCAAGTGATGAAGC
	R: GAGGAACTGCACACAATCGC
OSBPL6	F: CTGTCGGTGCTGGAGATCG
	R: CTGTTTTCCCAGCAAGTGGC
IGFBP3	F: ATGCTAGTGAGTCGGAGGAA
	R: ATTCTGTCTCCCGCTTGGAC
GADD45B	F: TCTGCCTCTTGGCCATTGAC
	R: CCGTGTGAGGGTTCGTGAC
GDF15 ChIP	F: GCCAGCTGAGGATGACGATT
	R: CACGCTCGCTCCCATCATAA

siRNA	
Name	Sequence
CXXC4-1#	S: GGGAAUGCAUGAACAAGCUTT
	AS: AGCUUGUUCAUGCAUUCCCTT
CXXC4-2#	S: CACAGACAGUGCGUUUCAATT
	AS: UUGAAACGCACUGUCUGUGTT
GDF15-1#	S: CCAGCUACAAUCCCAUGGUTT
	AS: ACCAUGGGAUUGUAGCUGGTT
GDF15-2#	S: CCGGAUACUCACGCCAGAATT
	AS: UUCUGGCGUGAGUAUCCGGTT
Sp1-1#	S: GUGCAAACCAACAGAUUAUTT
	AS: AUAAUCUGUUGGUUUGCACTT
Sp1-2#	S: CCAGCAACAUGGGAAUUAUTT
	AS: AUAAUUCCCAUGUUGCUGGTT

F: Forward Primer.

R: Reversed Primer.

S: Sense.

AS: Anti-sense.

### Small interfering RNAs (siRNAs) and transfection

siRNAs specific for CXXC4, GDF15 and Sp1 were synthesized by GenePharma (Shanghai, China). The sequences of siRNAs are listed in Table 1. Cells were seeded over-night in 6-well plates (4*10^5^/well) and transfected with siRNA duplexes (20 nM) using LipofectamineTM RNAiMAX transfection reagent (Invitrogen, Carlsbad, CA, USA) according to the manufacturer’s instructions.

### Chromatin immunoprecipitation assay

ChIP assay was carried out using the SimpleChIPTM Enzymatic Chromatin IP Kit (Cell Signaling Technology, Danvers, MA, USA). In brief, 4*10^7^ cells were fixed with 4% formaldehyde. Cells were then lysed and chromatin was harvested and fragmented using enzyme digestion. The chromatin was then subjected to immunoprecipitation using antibodies specific to CXXC4 or Sp1. After immunoprecipitation, the protein-DNA cross-links were reversed and the DNA was purified. The enriched DNA sequence was detected by quantitative real-time PCR with a pair of primers around the CXXC4 binding sites of GDF15. The primers used for ChIP assay are listed in Table 1.

### Statistical analysis

The Student’s t-test was used to explore the difference in gene expression and cell growth. The probability of overall survival was calculated with the Kaplan–Meier method. All statistical analyses were performed using Graph-Pad Prism or SPSS software 22.0. P < 0.05 was considered statistically significant.
